# Long-term Parkinson’s disease quality of life after staged DBS: STN vs GPi and first vs second lead

**DOI:** 10.1038/s41531-020-0115-3

**Published:** 2020-07-06

**Authors:** Stephanie Cernera, Robert S. Eisinger, Joshua K. Wong, Kwo Wei David Ho, Janine Lobo Lopes, Kevin To, Samuel Carbunaru, Adolfo Ramirez-Zamora, Leonardo Almeida, Kelly D. Foote, Michael S. Okun, Aysegul Gunduz

**Affiliations:** 1 J. Crayton Pruitt Department of Biomedical Engineering, Gainesville, FL USA; 2 Department of Neuroscience, Norman Fixel Institute for Neurological Diseases, Gainesville, FL USA; 3 Department of Neurology, Norman Fixel Institute for Neurological Diseases, Gainesville, FL USA; 4 Department of Neurosurgery, Norman Fixel Institute for Neurological Diseases, Gainesville, FL USA

**Keywords:** Parkinson's disease, Parkinson's disease

## Abstract

Deep brain stimulation (DBS) for Parkinson’s disease (PD) improves quality of life (QoL), but longitudinal follow-up data are scarce. We sought to quantify long-term benefits of subthalamic nucleus (STN) vs globus pallidus internus (GPi), and unilateral vs staged bilateral PD-DBS on postoperative QoL. This is a retrospective, longitudinal, non-randomized study using the PD QoL questionnaire (PDQ)-39 in patients with STN- or GPi-DBS, and with unilateral (*N* = 191) or staged bilateral (an additional contralateral lead implant) surgery (*N* = 127 and 156 for the first and second lead, respectively). Changes in PDQ-39 summary index (PDQ-39SI) and subscores throughout 60 months of follow-up were used as the primary analysis. We applied mixed models that included levodopa and covariates that differed at baseline across groups. For unilateral implantation, we observed an initial improvement in PDQ-39SI of 15.55 ± 3.29% (µ ± SE) across both brain targets at 4 months postoperatively. Unilateral STN patients demonstrated greater improvement in PDQ-39SI than GPi patients at 4 and 18 months postoperatively. Analysis of patients with staged bilateral leads revealed an initial 25.34 ± 2.74% (µ ± SE) improvement in PDQ-39SI at 4 months after the first lead with further improvement until 18 months, with no difference across targets. Scores did not improve after the second lead with gradual worsening starting at 18 months postoperatively. STN-DBS provided greater short-term QoL improvement than GPi-DBS for unilateral surgery. For staged bilateral DBS, overall QoL improvement was explained primarily by the first lead. Decision-making for patients considering DBS should include a discussion surrounding the potential risks and benefits from a second DBS lead.

## Introduction

Quality of life (QoL) is one of the most important outcome measures in healthcare^1,2^. Parkinson’s disease (PD) patients experience a multitude of motor and non-motor symptoms, resulting in a debilitating reduction in QoL^3,4^. Today, deep brain stimulation (DBS) of the subthalamic nucleus (STN) or globus pallidus internus (GPi) is considered a safe and effective surgical treatment for PD based on randomized controlled trials that have included motor symptom scales and QoL as primary or secondary outcomes^5–9^. In PD, QoL is commonly assessed using the validated PD QoL questionnaire (PDQ)-39^10^. Although DBS has been shown to improve QoL, longitudinal QoL follow-up data after PD-DBS are scarce and are mostly drawn from databases with small sample sizes or limited target inclusion^11^.

Since GPi stimulation is becoming increasingly used for PD-DBS^6,12–15^, data are needed that compare not only the effects of STN or GPi stimulation on QoL measurements, but also of unilateral and staged bilateral stimulation in each of these brain targets. Furthermore, current studies of QoL outcomes with bilateral implants have reported outcomes before and after the implantation of both leads^5,6,16,17^; however, data are lacking that systematically analyze the QoL effects of each lead independently. With real-life data from standard-of-care intervention, we aimed to evaluate the long-term effects of DBS on QoL across both brain targets and surgery types, while also uncovering the effects of the first vs the second lead in patients with staged bilateral implantations.

## Results

### Sample characteristics

A total of 121 unilateral GPi and 70 unilateral STN patients were included. For bilateral surgery, 60 GPi and 67 STN patients were included for the first lead, and 72 GPi and 84 STN patients were included for the second lead. Baseline PDQ scores for unilateral, bilateral first, and bilateral second surgeries were obtained 4.91 ± 0.20, 4.99 ± 0.20, and 2.48 ± 0.21 months before surgery. At baseline, unilateral STN patients had a higher tremor score compared to GPi patients (*p* < 0.01), and unilateral GPi patients had a worse postural instability gait disorder (PIGD) score compared to STN patients (*p* < 0.01; Table 1, Supplementary Table 1). Therefore, in our unilateral analyses, we included tremor score, PIGD, an interaction between tremor score and target, and an interaction between PIGD and target to control for the influence of these differences on QoL outcomes. For the PDQ mobility subscore specifically, baseline mobility scores were added as a covariate since STN patients had a lower baseline score compared to GPi patients (*p* < 0.05; Table 1, Supplementary Table 1).

**Table 1:** Baseline characteristics for unilateral patients^a,b^.

Variable (mean ± SD (*N*))	STN (70)	GPi (121)	STN vs GPi
Age	65.07 ± 7.88 (70)	66.09 ± 7.71 (120)	*p* = 0.39
Age at disease onset	53.57 ± 10.23 (65)	54.08 ± 9.40 (102)	*p* = 0.99
Disease duration	11.51 ± 6.20 (65)	11.83 ± 5.69 (102)	*p* = 0.72
Gender (male no./no.)	57/70	85/120	*p* = 0.15
UPDRS-I	2.81 ± 2.35 (63)	2.49 ± 1.98 (108)	*p* = 0.53
UPDRS-II	15.91 ± 5.96 (64)	15.73 ± 6.17 (111)	*p* = 0.85
UPDRS-III off-med	37.34 ± 11.78 (65)	38.27 ± 11.03 (114)	*p* = 0.60
UPDRS-III off-med contralateral tremor	4.15 ± 2.46 (65)	3.09 ± 2.16 (114)	***p*** < **0.01**
UPDRS-III off-med contralateral rigidity	3.45 ± 1.53(65)	3.57 ± 1.44 (114)	*p* = 0.77
UPDRS-III off-med contralateral bradykinesia	7.52 ± 2.62 (65)	7.50 ± 2.56 (114)	*p* = 0.96
UPDRS-III off-med PIGD	4.29 ± 2.62 (65)	5.42 ± 3.07 (114)	***p*** < **0.01**
LEDD	1439 ± 936 (68)	1407 ± 822 (118)	*p* = 0.85
Berg balance scale	51.65 ± 2.91 (26)	50.30 ± 5.29 (73)	*p* = 0.37
TUG	9.55 ± 1.83 (31)	10.89 ± 11.83 (79)	*p* = 0.55
Dopamine responsiveness^c^	−32.32 ± 23.89 (62)	−36.24 ± 18.03 (109)	*p* = 0.46
MMSE	28.04 ± 2.10 (24)	28.67 ± 1.73 (30)	*p* = 0.19
SWAL-QOL	85.10 ± 10.91 (19)	80.56 ± 11.27 (24)	*p* = 0.17
Anxiety	17.67 ± 7.04 (12)	15.00 ± 9.53 (40)	*p* = 0.24
Depression	10.52 ± 6.43 (31)	11.04 ± 7.45 (76)	*p* = 0.95
Total PDQ	30.80 ± 15.25 (70)	31.73 ± 15.22 (121)	*p* = 0.47
Mobility	36.34 ± 23.02 (70)	44.49 ± 27.89 (121)	***p*** < **0.05**
ADL	39.29 ± 21.41 (70)	36.00 ± 21.66 (121)	*p* = 0.32
Emotional well-being	29.46 ± 19.21 (70)	30.11 ± 20.22 (121)	*p* = 0.85
Stigma	30.00 ± 26.03 (70)	25.57 ± 22.20 (121)	*p* = 0.34
Social support	10.65 ± 13.69 (70)	15.98 ± 19.58 (121)	*p* = 0.17
Cognition	28.31 ± 18.33 (70)	29.11 ± 20.14 (121)	*p* = 0.95
Communication	27.02 ± 21.31 (70)	29.99 ± 21.16 (121)	*p* = 0.34
Bodily discomfort	45.36 ± 22.04 (70)	42.29 ± 22.84 (121)	*p* = 0.44

^a^All statistics can be found in Supplementary Table 1.

^b^Bold values represent significant differences.

^c^A negative value indicates more dopamine responsiveness.

In the staged bilateral cohort, at the first surgery, the STN cohort exhibited significantly higher scores on the Berg balance scale (*p* < 0.05) and lower PIGD scores (*p* < 0.05) than the GPi cohort (Table 2, Supplementary Table 2). Furthermore, three significantly different baseline variables between the staged bilateral STN and GPi cohorts were present at the second lead surgery, namely rigidity (*p* < 0.05), levodopa equivalent dose (LEDD; *p* < 0.05), and PIGD (*p* < 0.001), which were all lower in the STN cohort. Additionally, in the STN group, patients showed more dopamine responsiveness (*p* < 0.01), a higher rigidity score (*p* < 0.01), a higher unified Parkinson’s disease rating scale (UPDRS)-II score (*p* < 0.05), and higher LEDD use (*p* < 0.01) before the first compared to the second lead. Furthermore, in the GPi group, patients had higher UPDRS-II (*p* < 0.05) and tremor scores (*p* < 0.05) before the first compared to the second lead. The number of months between the first and second surgeries was similar for the GPi and STN groups (*p* = 0.27; Table 2, Supplementary Table 2).

**Table 2:** Baseline characteristics for bilateral patients^a,b^.

Variable (mean ± SD (*N*))	Bilateral first lead			Bilateral second lead			Bilateral first vs second	
	STN (67)	GPi (60)	STN vs GPi	STN (84)	GPi (72)	STN vs GPi	STN	GPi
Age	61.91 ± 8.82 (67)	63.95 ± 8.56 (60)	*p* = 0.19	62.73 ± 9.53(84)	64.79 ± 8.37 (72)	*p* = 0.24	*p* = 0.42	*p* = 0.57
Age at disease onset	50.46 ± 9.10 (63)	50.31 ± 9.54 (49)	*p* = 0.93	50.87 ± 9.63 (77)	50.42 ± 9.45 (59)	*p* = 0.78	*p* = 0.80	*p* = 0.95
Disease duration	11.11 ± 4.59 (63)	12.39 ± 4.91(49)	*p* = 0.19	11.83 ± 4.54 (77)	13.49 ± 5.15 (59)	*p* = 0.052	*p* = 0.27	*p* = 0.26
Months between surgeries	–	–	–	14.75 ± 13.30 (84)	11.87 ± 11.01 (72)	*p* = 0.27	–	–
Gender (male no./no.)	52/67	46/60	*p* = 0.90	65/84	43/72	*p* = 0.58	*p* = 0.97	*p* = 0.69
LEDD	1572 ± 1443 (67)	1343 ± 733 (60)	*p* = 0.46	1119 ± 756 (84)	1559 ± 1921 (72)	***p*** < **0.05**	***p*** < **0.01**	*p* = 0.66
UPDRS-I	2.95 ± 2.05 (60)	2.25 ± 1.93 (56)	*p* = 0.054	2.43 ± 1.80 (80)	2.11 ± 1.81 (69)	*p* = 0.15	*p* = 0.15	*p* = 0.71
UPDRS-II	15.95 ± 5.91 (59)	16.66 ± 5.54 (56)	*p* = 0.51	13.28 ± 5.52 (79)	14.36 ± 5.33 (69)	*p* = 0.23	***p*** < **0.01**	***p*** < **0.05**
UPDRS-III off-med	41.17 ± 11.91 (58)	40.71 ± 10.94 (59)	*p* = 0.83	38.54 ± 10.80 (59)	40.67 ± 10.63 (58)	*p* = 0.19	*p* = 0.15	*p* = 0.98
UPDRS-III off-med contralateral tremor	4.00 ± 2.58 (58)	3.49 ± 2.41 (59)	*p* = 0.31	3.44 ± 2.48 (59)	2.66 ± 2.50 (58)	*p* = 0.07	*p* = 0.24	***p*** < **0.05**
UPDRS-III off-med contralateral rigidity	3.71 ± 1.53 (58)	3.36 ± 1.48 (59)	*p* = 0.30	3.07 ± 1.40 (59)	3.84 ± 1.71 (58)	***p*** < **0.05**	***p*** < **0.05**	*p* = 0.24
UPDRS-III off-med contralateral bradykinesia	8.05 ± 2.69 (58)	7.59 ± 2.61 (59)	*p* = 0.32	7.47 ± 2.43 (59)	8.00 ± 2.82 (58)	*p* = 0.28	*p* = 0.23	*p* = 0.46
UPDRS-III off-med PIGD	4.07 ± 2.38 (58)	5.44 ± 3.02 (59)	***p*** < **0.05**	4.00 ± 2.26 (59)	5.79 ± 3.08 (58)	***p*** < **0.001**	*p* = 0.84	*p* = 0.53
Berg balance scale	52.58 ± 4.10 (12)	48.67 ± 6.39 (30)	***p*** < **0.05**	50.08 ± 7.27 (26)	49.51 ± 5.15 (39)	*p* = 0.19	*p* = 0.23	*p* = 0.79
TUG	8.54 ± 2.60 (14)	9.22 ± 4.08 (31)	*p* = 0.71	12.62 ± 15.17 (25)	9.96 ± 3.87 (45)	*p* = 0.57	*p* = 0.43	*p* = 0.15
Dopamine responsiveness^c^	−39.11 ± 22.31 (57)	−34.45 ± 19.51 (57)	*p* = 0.08	−30.99 ± 18.04 (54)	−30.05 ± 17.48 (53)	*p* = 0.78	***p*** < **0.01**	*p* = 0.21
MMSE	29.05 ± 0.97 (21)	27.75 ± 3.82 (20)	*p* = 0.50	–	–	–	–	–
SWAL-QOL	85.80 ± 9.58 (10)	86.74 ± 7.62 (17)	*p* = 0.79	88.86 ± 10.86 (14)	83.95 ± 10.21 (21)	*p* = 0.08	*p* = 0.34	*p* = 0.60
Anxiety	12.00 ± 8.09 (23)	13.25 ± 9.05 (12)	*p* = 0.98	12.29 ± 8.05 (66)	15.00 ± 9.41 (54)	*p* = 0.12	*p* = 0.58	*p* = 0.59
Depression	10.79 ± 6.46 (14)	8.00 ± 6.91 (36)	*p* = 0.44	7.79 ± 6.19 (34)	8.41 ± 6.57 (56)	*p* = 0.64	*p* = 0.10	*p* = 0.45
Total PDQ	31.72 ± 14.87 (67)	32.79 ± 14.68 (60)	*p* = 0.69	23.70 ± 12.51 (84)	25.87 ± 11.97 (72)	*p* = 0.18	***p*** < **0.001**	***p*** < **0.01**
Mobility	40.82 ± 26.76 (67)	49.12 ± 25.58 (60)	*p* = 0.06	34.00 ± 24.14 (84)	39.70 ± 21.12 (72)	*p* = 0.08	*p* = 0.13	***p*** < **0.05**
ADL	43.66 ± 23.66 (67)	41.60 ± 23.01 (60)	*p* = 0.64	29.94 ± 18.59 (84)	31.80 ± 18.65 (72)	*p* = 0.47	***p*** < **0.001**	***p*** < **0.05**
Emotional well-being	30.85 ± 22.41 (67)	28.02 ± 21.08 (60)	*p* = 0.52	20.41 ± 16.80 (84)	19.65 ± 16.67 (72)	*p* = 0.88	***p*** < **0.01**	***p*** < **0.05**
Stigma	26.26 ± 23.35 (67)	24.48 ± 22.64 (60)	*p* = 0.65	12.95 ± 14.93 (84)	19.75 ± 18.56 (72)	***p*** < **0.05**	***p*** < **0.001**	*p* = 0.33
Social support	16.48 ± 19.33 (67)	14.51 ± 19.55 (60)	*p* = 0.39	9.97 ± 14.58 (84)	11.46 ± 17.28 (72)	*p* = 0.51	***p*** < **0.05**	*p* = 0.34
Cognition	28.17 ± 17.82 (67)	27.55 ± 18.44 (60)	*p* = 0.69	22.92 ± 15.48 (84)	21.40 ± 14.86 (72)	*p* = 0.53	***p*** < **0.05**	*p* = 0.07
Communication	28.54 ± 18.67 (67)	29.86 ± 22.01 (60)	*p* = 0.97	24.31 ± 17.85 (84)	22.86 ± 17.09 (72)	*p* = 0.65	*p* = 0.16	*p* = 0.11
Bodily discomfort	38.99 ± 22.64 (67)	47.01 ± 21.55 (60)	***p*** < **0.05**	31.80 ± 22.67 (84)	39.41 ± 20.73 (72)	***p*** < **0.01**	***p*** < **0.05**	***p*** < **0.05**

^a^All statistics can be found in Supplementary Table 2.

^b^Bold values represent significant differences.

^c^A negative value indicates more dopamine responsiveness.

Given these numerous differences across the staged bilateral surgery cohorts, mixed models included the following covariates: rigidity score, UPDRS-II, tremor score, PIGD, Berg balance scale, LEDD, and dopamine responsiveness, as well as interactions between rigidity score and target; rigidity score and first vs second surgery; UPDRS-II and first vs second surgery; tremor score and first vs second surgery; PIGD and target; Berg balance scale and target; LEDD and target; LEDD and first vs second surgery; and dopamine responsiveness and first vs second surgery. PDQ-39 summary index (PDQ-39SI) and all PDQ subscores except communication demonstrated baseline differences with respect to target, the first vs second surgery, or both (Table 2, Supplementary Table 2). These baseline scores and the necessary interactions were thus also added to each mixed model when appropriate.

### Unilateral Implantation

**Fig. 1:**
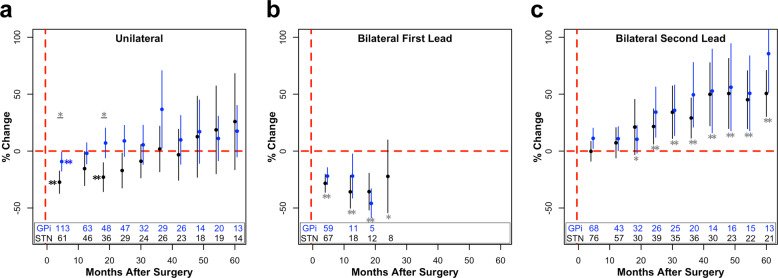
Percent change in PDQ-39SI across surgical cohorts. Percent change (µ ± 2 × s.e.m.) in PDQ-39SI after unilateral DBS (**a**), after the first lead of staged bilateral DBS (**b**), and after the second lead of staged bilateral DBS (**c**) of the GPi (blue) or STN (black). The number of patients included in each data point across time is listed at the bottom of each graph. For visualization purposes, bins with less than five data points are removed. Blue (GPi) or black (STN) stars (*p < 0.05; **p < 0.01) represent significant improvements in PDQ scores compared to baseline values, whereas gray stars with a line underneath represent significant differences between the STN and GPi at the specified time point. If a target was not significant within the mixed model, STN and GPi were pooled together, and compared to baseline, represented by a gray star without a line (*p < 0.05; **p < 0.01). These values were corrected for multiple comparisons using false discovery rate correction.

**Fig. 2:**
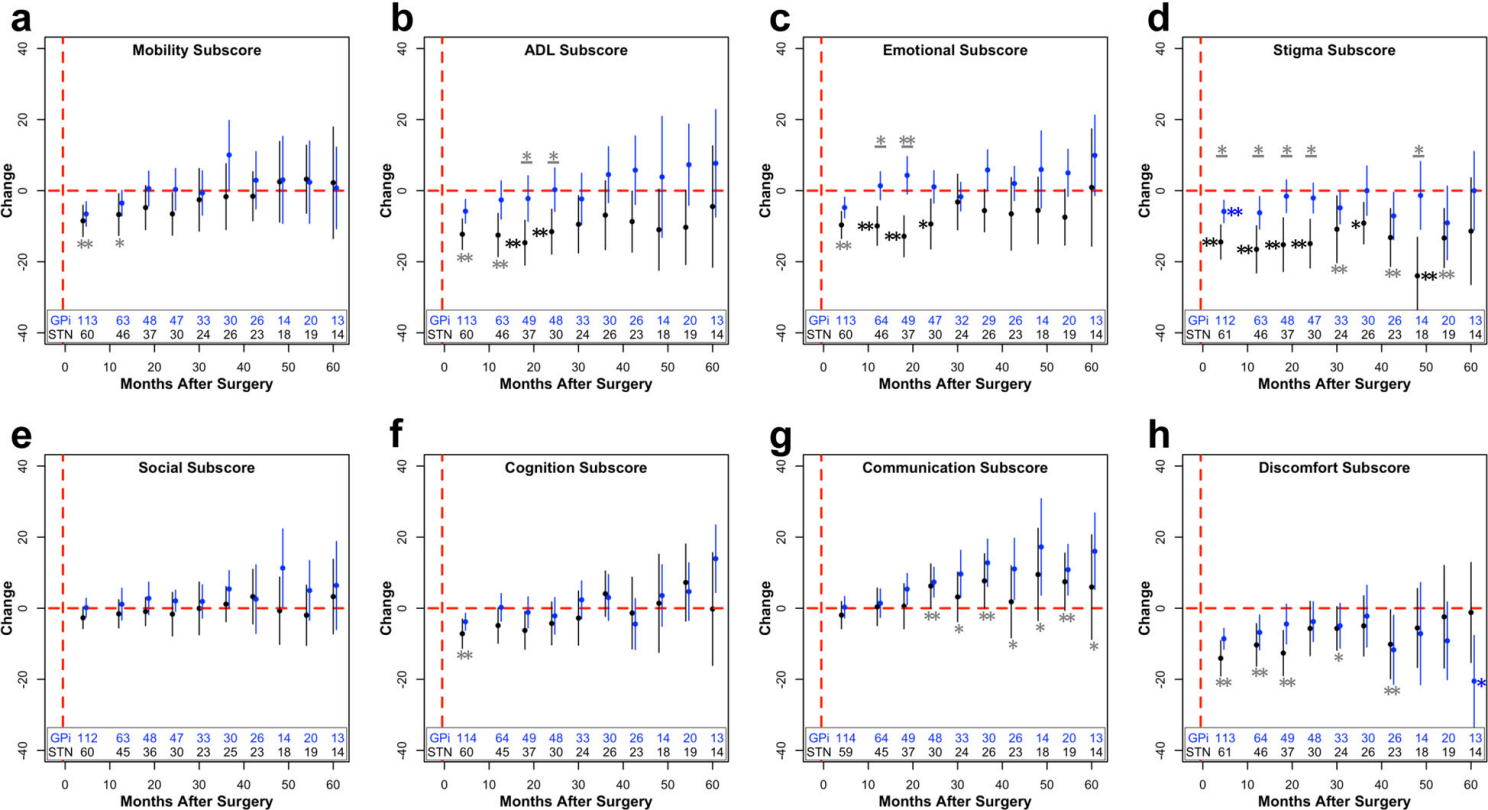
Change in subscores of PDQ after unilateral surgery. Change from baseline (µ ± 2 × s.e.m.) within the mobility (**a**), ADL (**b**), emotional well-being (**c**), stigma (**d**), social support (**e**), cognition (**f**), communication (**g**), and bodily discomfort (**h**) subscores after unilateral surgery. The number of patients included in each data point across time is listed at the bottom of each graph. For visualization purposes, bins with less than five data points are removed. Blue (GPi) or black (STN) stars (*p < 0.05; **p < 0.01) represent significant improvements in PDQ scores compared to baseline values, whereas gray stars with a line underneath represent significant differences between the STN and GPi at the specified time point. If a target was not significant within the mixed model, STN and GPi were pooled together, and compared to baseline, represented by a gray star without a line. These values were corrected for multiple comparisons using false discovery rate correction.

For unilateral implantation, we observed a significant main effect of time (*p* < 0.0001) and target (*p* < 0.05) on postoperative percent change in PDQ-39SI (Fig. 1a). Across targets compared to baseline, there was an initial improvement in PDQ-39SI at 4 months, whereas PDQ-39SI scores gradually returned to baseline during the remainder of follow-up (Fig. 1a). Regarding the effect of target, STN patients showed greater improvement in PDQ-39SI compared to GPi patients, particularly at 4 (27.32 ± 4.94% vs 9.20 ± 4.20%, *p* < 0.05) and 18 (22.92 ± 6.36% vs 7.12 ± 6.57%, *p* < 0.05) months of follow-up (Fig. 1a). Similar to PDQ-39SI, for all unilateral subscore mixed models (Fig. 2), there was also a significant main effect of time (Table 3), with the exception of the stigma subscore. In the cases of the ADL (*p* < 0.01) and communication (*p* < 0.05) subscores, STN patients improved more and worsened less, respectively, compared to GPi patients and independent of other covariates (Fig. 2, Table 3). Postoperative improvement was associated with increased LEDD for mobility (*p* < 0.05) and ADL (*p* < 0.001) subscores. Additionally, we observed positive associations between baseline tremor severity and postoperative improvement in the ADL (*p* < 0.05) and stigma (*p* < 0.05) subscores, as well as between baseline mobility scores and postoperative improvement in mobility (*p* < 0.0001). Lastly, for STN and GPi patients, higher PIGD scores at baseline were associated with less and more improvement in the ADL subscore, respectively (Supplementary Table 3).

**Table 3:** Unilateral mixed model results^a,b^.

	Time d.f. = 9	Target d.f. = 1	LEDD d.f. = 1	Tremor score d.f. = 1	Baseline score d.f. = 1	PIGD × target d.f. = 1
	*F*, *p*	*F*, *p*	*F*, *p*	*F*, *p*	*F*, *p*	*F*, *p*
Total	**10.19**, <**0.0001**	**5.57**, <**0.05**	2.20, 0.14	0.77, 0.38	NA	3.89, 0.0502
Mobility	**5.87**, <**0.0001**	2.12, 0.15	**5.97**, <**0.05**	1.78, 0.18	**21.56**, <**0.0001**	2.32, 0.13
ADL	**3.85**, <**0.001**	**9.42**, <**0.01**	**14.49**, <**0.001**	**5.92**, <**0.05**	NA	**11.44**, <**0.001**
Emotional well-being	**4.21**, <**0.001**	3.44, 0.07	0.58, 0.45	007, 0.79	NA	0.17, 0.68
Stigma	1.75, 0.08	3.69, 0.06	0.30, 0.58	**4.34**, <**0.05**	NA	3.58, 0.06
Social support	**3.57**, <**0.001**	1.81, 0.18	0.26, 0.61	0.03, 0.87	NA	0.01, 0.93
Cognition	**6.14**, <**0.0001**	0.01, 0.94	0.78, 0.38	0.07, 0.79	NA	0.60, 0.43
Communication	**9.29**, <**0.0001**	**5.78**, <**0.05**	3.34, 0.07	0.05, 0.83	NA	1.56, 0.21
Bodily discomfort	**2.98**, <**0.01**	0.03, 0.88	0.00, 0.99	2.56, 0.11	NA	0.09, 0.76

^a^All main effects of PIGD, time × target interactions, baseline score × target, and tremor score × target interactions were insignificant and are not included in this table.

^b^Bold values represent significant effects.

### Staged bilateral implantation

Within the bilateral lead implantation cohort, there was a significant effect of time (*p* < 0.01) and first vs second lead surgery (*p* < 0.05; Table 4), suggesting an overall improvement in PDQ after the first (*p* < 0.0001), but overall worsening after the second (*p* < 0.0001) lead placement across all follow-up. More specifically, for the first lead implantation across targets, there was an initial large improvement in PDQ-39SI within 18 months postoperatively (Fig. 1b). For the second lead implantation, PDQ-39SI returned to baseline by 4 months and progressively worsened starting at 18 months postoperatively (Fig. 1c). These results suggest that at the sample level, the second lead provided sustained (but not additional) benefit beyond the first lead for at least 12 months.

**Table 4:** Bilateral mixed model results^a,b^.

	Time d.f. = 9	First vs second surgery d.f. = 1	Baseline scores d.f. = 1	Dopamine responsiveness d.f. = 1	LEDD d.f. = 1	UPDRS-II d.f. = 1	Tremor score d.f. = 1	Baseline × first vs second surgery d.f. = 1	Rigidity score × target d.f. = 1	LEDD × first vs second surgery d.f. = 1	LEDD × target d.f. = 1	Tremor score × first vs second surgery d.f. = 1
	*F*, *p*	*F*, *p*	*F*, *p*	*F*, *p*	*F*, *p*	*F*, *p*	*F*, *p*	*F*, *p*	*F*, *p*	*F*, *p*	*F*, *p*	*F*, *p*
Total	**2.85**, <**0.01**	**4.28**, <**0.05**	**11.19**, <**0.01**	0.08, 0.77	**13.26**, <**0.001**	0.26, 0.61	0.05, 0.83	0.44, 0.51	1.67, 0.20	**5.30**, <**0.05**	**6.86**, <**0.01**	**4.28**, <**0.05**
Mobility	1.10, 0.37	1.27, 0.26	**64.99**, <**0.0001**	0.10, 0.76	3.44, 0.07	1.00, 0.32	0.03, 0.87	1.48, 0.23	**4.93**, <**0.05**	**4.23**, <**0.05**	0.03, 0.86	0.46, 0.50
ADL	1.61, 0.12	0.11, 0.74	**26.00**, <**0.0001**	2.08, 0.15	2.09, 0.09	**7.02**, <**0.01**	**6.69**, <**0.05**	**9.32**, <**0.01**	2.31, 0.13	**4.55**, <**0.05**	0.16, 0.69	0.92, 0.34
Emotional well-being	1.92, 0.06	1.95, 0.16	**21.68**, <**0.0001**	0.04, 0.85	**4.00**, <**0.05**	0.30, 0.58	0.18, 0.67	**6.51**, <**0.05**	0.09, 0.77	1.59, 0.21	0.04, 0.84	**9.89**, <**0.01**
Stigma	0.65, 0.75	0.04, 0.84	**13.34**, <**0.001**	0.99, 0.32	1.85, 0.18	0.13, 0.72	1.14, 0.29	0.15, 0.70	2.36, 0.13	0.41, 0.53	0.39, 0.53	0.40, 0.53
Social support	1.29, 0.25	0.10, 0.75	**15.39**, <**0.001**	0.45, 0.51	0.30, 0.59	0.13, 0.72	0.11, 0.74	3.36, 0.07	0.28, 0.60	0.06, 0.81	0.16, 0.69	0.01, 0.93
Cognition	1.63, 0.11	0.51, 0.48	**11.04**, <**0.01**	**4.44**, <**0.05**	2.34, 0.13	0.27, 0.61	0.34, 0.56	**22.96**, <**0.0001**	1.30, 0.26	0.05, 0.82	3.14, 0.08	3.64, 0.06
Communication	1.03, 0.42	0.02, 0.89	NA	1.07, 0.30	**5.91**, <**0.05**	1.17, 0.28	0.24, 0.62	NA	0.09, 0.76	2.88, 0.09	2.66, 0.11	2.82, 0.10
Bodily discomfort	0.58, 0.81	0.44, 0.51	**9.46**, <**0.01**	2.19, 0.14	2.18, 0.14	1.09, 0.30	3.38, 0.07	1.93, 0.17	0.29, 0.59	0.20, 0.66	3.75, 0.06	2.66, 0.11

^a^All main effects of rigidity score, PIGD, Berg balance scale, time × target, baseline score × target, rigidity score × first vs second surgery, UPDRS-II × first vs second surgery, PIGD × target, Berg balance scale × target, dopamine responsiveness × target, tremor score × target, and time × target × first vs second surgery interactions were insignificant and are not included in this table.

^b^Bold values represent significant effects.

Postoperative medication (med) adjustments were related to changes in PDQ. Namely, the effect of the first vs the second surgery (*p* < 0.05) and target (*p* < 0.01) for PDQ-39SI outcomes further interacted with LEDD. Specifically, a greater postoperative decrease in LEDD was associated with more improvement after the first vs the second surgery, as well as in the STN vs the GPi cohort (Supplementary Table 3). For both the mobility (*p* < 0.05) and ADL (*p* < 0.05) subscores, a postoperative decrease in LEDD was associated with more improvement after the first surgery (Supplementary Table 3). For the subscores emotional well-being (*p* < 0.05) and communication (*p* < 0.05), postoperative med reduction was linked to postoperative improvement independent of the first vs the second surgery and target (Table 4). Lastly, more baseline dopamine responsiveness was associated with postoperative worsening of the cognition subscore, independent of target (*p* < 0.05; Table 4).

Excluding communication, all PDQ models included baseline PDQ scores as a covariate. In all cases, higher baseline PDQ subscores were significantly associated with more postoperative improvement (Table 4). In addition, this relationship was stronger after the first surgery compared to the second surgery for the ADL (*p* < 0.01), emotional well-being (*p* < 0.05), and cognition subscores (*p* < 0.001; Supplementary Table 3).

There were a few other notable dependencies of our model variables on the change in PDQ after staged bilateral surgery (Supplementary Tables 3 and 4). Higher baseline tremor scores were associated with less improvement in emotional well-being after the first surgery (*p* < 0.01). Higher baseline tremor scores were also associated with more postoperative improvement in the ADL subscore (*p* < 0.05), independent of the first vs the second surgery and of target. Finally, independent of the surgery, the ADL subscore further depended on UPDRS-II, in which higher scores were associated with postoperative improvement (*p* < 0.01).

## Discussion

Although DBS alleviates motor symptoms associated with PD, the long-term effect of DBS on QoL has not been established largely because long-term longitudinal QoL data after DBS are scarce. Furthermore, QoL data comparing targets (i.e., STN vs GPi) are lacking across different types of implantations (i.e., unilateral and staged bilateral). Furthermore, the effect of using a staged bilateral approach is less understood since most centers have traditionally performed simultaneous implantations. Addressing these gaps in knowledge, overall we found that STN-DBS offered a short-term QoL benefit superior to GPi-DBS for unilateral surgery, but with no long-term differences. The data also informed us that the majority of overall QoL improvement after staged bilateral DBS was explained by the first lead.

In our unilateral outcome analyses, PDQ-39SI significantly improved regardless of brain target, but more improvement was seen with STN implantation (Table 3). Although improvement varied across subscores, PDQ generally improved in the short-term and stabilized in the long-term. No short-term or long-term improvements were found in the domains of communication and social support (Fig. 2). This is consistent with literature suggesting that speech does not typically respond to DBS^18–21^. Furthermore, the microlesion effect from surgery may impair verbal fluency^22–24^, and there remains ongoing progression of PD after DBS^25^. A lack of any significant change in the social support subscore may reflect our extensive preoperative neuropsychological screening, and the availability or quality of social support^26^.

Beyond the choice of brain target, several covariates significantly and independently affected postoperative PDQ outcomes after unilateral DBS. More LEDD use was associated with improvements in mobility and ADL subscores, which likely capture levodopa-responsive motor symptoms. Given the debilitating effects of tremor particularly on both ADLs and on stigma^27,28^, it was unsurprising that higher baseline tremor scores were associated with postoperative improvements for these subscores. A more unexpected result after unilateral DBS was that higher baseline PIGD scores were associated with less postoperative ADL improvement in the STN cohort, but more improvement in the GPi cohort. It is known that the ADL subscore is impacted by gait function^29^. DBS outcomes on gait have been highly heterogeneous^19,30^ and our results suggest that this could be in part due to differences across specific outcomes (e.g., different QoL domains), as well as a more complex interdependency between not only brain target, but also baseline patient status. Similarly, for mobility PDQ outcomes, patients with higher mobility subscores at baseline improved more after DBS. This may reflect a common observation in this study that patients with more motor deficits at baseline may perceive a larger additive benefit of DBS on their motor symptoms^31^.

In comparing these results to established literature, in a cohort of unilateral STN- and GPi-DBS patients Zahodne and colleagues^8^ reported improvements at 6 months in PDQ-39SI and in the subscores mobility, ADL, emotional well-being, stigma, cognition, and bodily discomfort, and these were overall similar to our results. However, for each target, they reported significant improvements in mobility, ADL, social support, and stigma for the GPi group, whereas the STN group only improved in stigma. In contrast, we found that the STN was superior in improving PDQ-39SI; however, our analyses were long-term. In addition, Zahodne et al. performed a randomized controlled trial, whereas this was a retrospective study. We did, however, attempt to minimize potential selection biases by controlling for many confounding clinical factors that differed at baseline.

Within the staged bilateral implantation cohort, after controlling for baseline PDQ scores, dopamine responsiveness, LEDD, UPDRS-II, tremor score, Berg balance scale, PIGD, and rigidity score, there was a significant, independent effect of time on postoperative PDQ-39SI. To our knowledge, this is the first study characterizing a larger overall improvement in PDQ-39SI from the first compared to the second surgery for staged bilateral patients. The second DBS lead provided sustained PDQ-39SI scores up to 12 months after surgery, followed by progressive worsening (Fig. 1c).

**Fig. 3:**
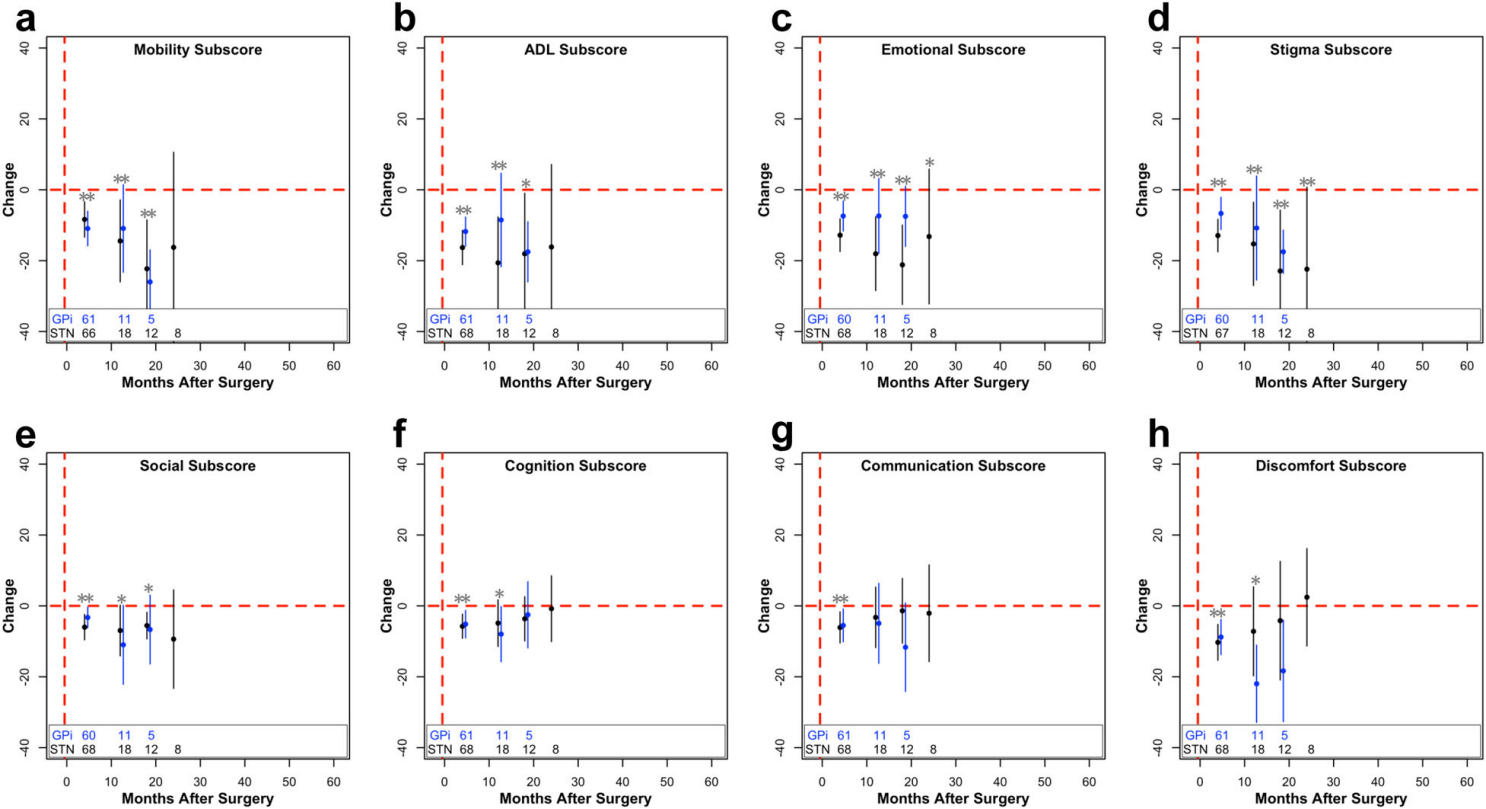
Change in subscores of PDQ after staged bilateral first surgery. Change from baseline (µ ± 2 × s.e.m.) within the mobility (**a**), ADL (**b**), emotional well-being (**c**), stigma (**d**), social support (**e**), cognition (**f**), communication (**g**), and bodily discomfort (**h**) subscores after staged bilateral first surgery. The number of patients included in each data point across time is listed at the bottom of each graph. For visualization purposes, bins with less than five data points are removed. Blue (GPi) or black (STN) stars (*p < 0.05; **p < 0.01) represent significant improvements in PDQ scores compared to baseline values, whereas gray stars with a line underneath represent significant differences between the STN and GPi at the specified time point. If a target was not significant within the mixed model, STN and GPi were pooled together, and compared to baseline, represented by a gray star without a line. These values were corrected for multiple comparisons using false discovery rate correction.

The first surgery may lead to larger improvements in QoL for several reasons (Fig. 1b, Fig. 3). This could be related to postoperative programming or med optimization that occurs between the two surgeries, though we included LEDD as a covariate in this model. Future studies could incorporate DBS programming settings or similar proxy measures, such as time until DBS optimization. Given the subjective nature of PDQ-39, it is also plausible that patient expectations differed from the first to the second surgery, and this could be an intriguing area for future studies^32,33^. The effect is likely not explained by asymmetric symptoms with respect to DBS laterality, since our analyses controlled for possible differences in contralateral motor scores between the first and second surgery. Emerging literature also has demonstrated bilateral effects from unilateral stimulation, suggesting the possibility for clinical improvement ipsilateral to the implanted lead^34–39^. Though our study cannot ascertain the causal explanation for this phenomenon, this result calls for a careful consideration about the necessity of a second lead, especially in patients with asymmetric symptoms, and highlights the importance of a careful risk–benefit analysis.

**Fig. 4:**
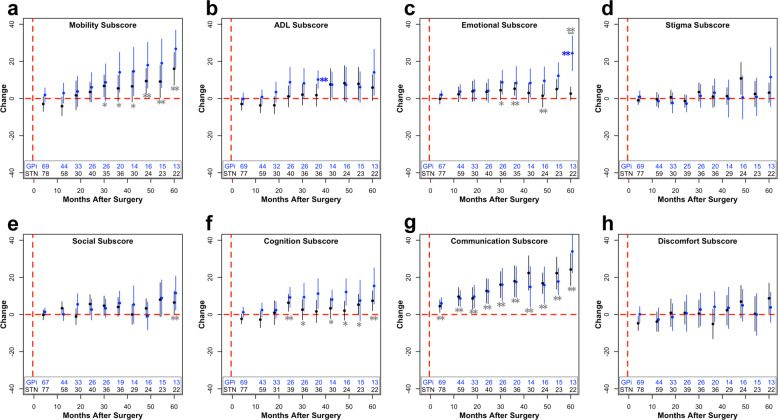
Change in subscores of PDQ after staged bilateral second surgery. Change from baseline (µ ± 2 × s.e.m.) within the mobility (**a**), ADL (**b**), emotional well-being (**c**), stigma (**d**), social support (**e**), cognition (**f**), communication (**g**), and bodily discomfort (**h**) subscores after staged bilateral second surgery. The number of patients included in each data point across time is listed at the bottom of each graph. For visualization purposes, bins with less than five data points are removed. Blue (GPi) or black (STN) stars (*p < 0.05; **p < 0.01) represent significant improvements in PDQ scores compared to baseline values, whereas gray stars with a line underneath represent significant differences between the STN and GPi at the specified time point. If a target was not significant within the mixed model, STN and GPi were pooled together, and compared to baseline, represented by a gray star without a line. These values were corrected for multiple comparisons using false discovery rate correction.

Within the literature, QoL outcomes after bilateral DBS have been characterized in randomized controlled trials, though not separated by first vs second lead implantation. In Follett and colleagues’ trial, QoL improved in six out of the eight subscales at 24 months after STN- or GPi-DBS, with communication worsening in both cohorts^6^. In our data, no statistical improvements were observed at 24 months in either the STN or GPi after the second lead implantation (Fig. 4). This difference in outcomes may be related to separating the first and second lead implantations. In the 36-month outcomes of the same study, patients were worse compared to baseline in only social support and cognition subscores^16^. In our data, across targets, patients were worse in the mobility, emotional well-being, and communication subscore. In ADL, patients in the GPi cohort worsened as well at 36 months. In another randomized controlled trial comparing STN and GPi outcomes at 12 and 36 months, there were no between group differences at either time point, which our results also demonstrated with the exception of the significant difference at 60 months in the emotional well-being subscore; however, the authors did not report individual PDQ subscores^14,40^. Overall, our data corroborate and extend prior literature findings.

All baseline PDQ scores except the communication subscore significantly affected postoperative change in PDQ. This finding was also stronger in the first vs the second surgery for the ADL, emotional well-being, and cognition subscores. This reinforces the need to control for baseline states in evaluating potential postoperative changes after DBS, and the importance of evaluating postoperative change with percentage change. As expected, patients with a worse baseline state improved more^31^, with the exception of communication, which could reflect the well-known lack of improvement in speech function from DBS^41^.

Similar to unilateral surgery, as expected, there was an important influence of LEDD on PDQ outcomes after staged bilateral DBS. We found that for bilateral surgery, increasing LEDD use was associated with less improvement in the first vs the second surgery for total PDQ-39SI, ADL, and mobility subscores, as well as for the STN vs the GPi cohorts for total PDQ-39SI. This effect was also found in the emotional well-being and communication subscores independent of target or the first vs second surgery. These effects could reflect a situation in which people with suboptimal DBS improvement tended to require more rescue LEDD as time progressed, and these individuals may have been more likely to progress to a contralateral implantation. Clearly, there is a complex interplay between DBS and LEDD, and to fully disentangle their relationship, a statistical model could include change scores for all covariates at all follow-ups to better track disease severity alongside changes in therapy.

The ADL subscore had several notable dependencies, specifically UPDRS-II and tremor scores. A higher baseline score for both variables was associated with postoperative improvements in the ADL subscore. The ADL subscore is correlated with UDPRS-II and UPDRS-III tremor scores^42,43^; thus, this outcome likely represents an improvement of ADL alongside lessening of tremor severity. Within the cognition subscore, more dopamine responsiveness was associated with postoperative worsening. The cognition subscore is more related to depression rather than cognitive functioning measured through neuropsychological exams^44^, and higher levodopa has been associated with a worsening of depression^45^. Finally, higher tremor scores were associated with less improvement in emotional well-being after the first surgery compared to the second. The emotional well-being subscore has been linked to mood measures, such as anxiety, depression, and apathy^44^; thus, this effect may stem from the functional impairments of tremor and their effects on mood^46^.

There are several limitations associated with these analyses. First, this was a retrospective study and data were limited to the scope of what was inputted into the University of Florida (UF) INFORM database. However, our data are representative of real-life outcomes and aimed to characterize current standard of care. The data also had a high dropout rate across time, which could influence our results compared to a completed dataset. We elected to not impute missing values due to potentially inaccurate data that may have been introduced to the model. Additionally, a UF-specific selection bias may have impacted STN vs GPi group effects. However, we controlled for a selection bias by including many baseline covariates. The way in which we binned and analyzed the data could also influence the results of the mixed model (Supplementary Fig. 1). Furthermore, it may be difficult to directly compare our results to the majority of studies examining DBS outcomes, using change—as opposed to percent change—in PDQ scores; however, as discussed, we opted to use percent change given the variability in baseline PDQ. Finally, our paper did not aim to directly explain why QoL changes after DBS surgery beyond associations of target and surgery type. Future studies could therefore develop statistical models using all covariates across time to better explain factors contributing most to QoL.

## Methods

### Study subjects

Data were retrospectively collected following Institutional Review Board approval to access the UF INFORM database from the Norman Fixel Institute for Neurological Diseases (IRB #201901807). All participants in the INFORM database provided written informed consent. PDQ-39 is routinely given for completion before clinical DBS programming visits. Inclusion criteria were unilateral and staged bilateral PD patients diagnosed by a movement disorders-trained neurologist, undergoing STN- or GPi-DBS. Staged bilateral cases were defined as bilateral lead implantations specifically in opposite hemispheres, in the same target, and on two different days. At UF, patients undergo a detailed risk–benefit profile assessment by an interdisciplinary team to determine their DBS candidacy^47,48^. Outcomes for bilateral patients were assessed for the first and second surgery, in which both interventions had different baseline assessments. Patients with only unilateral implantations were not included within the first bilateral implantation cohort. Due to separate analyses examining the effect of the first and second lead, and given that some patients lacked baseline scores for one but not both surgeries, different patients could be included in these two groups.

Baseline PDQ scores for all surgeries were defined as the score nearest the date of surgery, but no more than 12 months before the surgery. Only complete PDQ-39 questionnaires were included, and no data in this study was imputed. For bilateral cases, the baseline scores for the second lead implantation were obtained after the date of the first surgery. Similarly, follow-up assessments for the first lead were only considered if they occurred prior to the second lead implantation. Patients’ data were available up to 5 years after lead implantation.

The following potential confounding baseline parameters were included in the analysis: age at surgery, age at disease onset, disease duration, gender, LEDD, UPDRS-I, UPDRS-II, off-med UPDRS-III, contralateral off-med UPDRS-III rigidity score (referred to as rigidity score throughout), contralateral off-med UPDRS-III tremor score (referred to as tremor score throughout), contralateral off-med UPDRS-III bradykinesia score, UPDRS-III off-med PIGD score (sum of questions 27–30), Berg balance scale^49^, timed-up and go^50^, percent improvement from off- to on-med UPDRS-III (referred to as dopamine responsiveness throughout), mini-mental state examination (MMSE)^51^, swallowing quality of life (SWAL-QOL)^52–54^, Beck Anxiety Inventory (BAI)^55^, Beck Depression Inventory-II^56^, and baseline PDQ scores. We defined dopamine responsiveness as the difference between on-med and off-med UPDRS-III scores, divided by the off-med score. Therefore, negative values indicate improvement from medication (i.e., more dopamine responsiveness). UPDRS scores after DBS implantation were completed off-med and off-stimulation. Overall, these baseline measures were not available for all study participants (Tables 1 and 2).

### Data analysis

Changes from the baseline values were calculated in 4-month bins. Bins were centered at peaks in the distribution of the frequency of retrospective postoperative data (Supplementary Fig. 1). If a patient had multiple follow-up values within bins, we considered the mean value of the PDQ scores. For PDQ-39SI score, we used percent change from baseline, whereas for PDQ subscores, we used the difference from baseline, thus preventing undefined values from zeros present at baseline, which was not an issue encountered with PDQ-39SI.

### Statistical analysis

Potential differences in baseline scores across the groups were evaluated with unpaired *t*-test, Mann–Whitney *U*, or chi-squared analyses when appropriate. Normality was assessed using Shapiro–Wilk tests. Significant changes at follow-up were analyzed using mixed models to fully use the available data^57^. A term in the model for the random effect of each subject further allowed us to control for individual variability. We treated time as categorical bins since the time-dependent effect of DBS on PDQ may be nonlinear. Models were fitted using a restricted maximum likelihood estimation approach. For unilateral surgery, we tested for effects of target, time after surgery, and their interaction. For bilateral cases, the effects of target, time after surgery, first or second lead implantation, and their respective interactions were computed. Additionally, covariates stemming from baseline differences were included within mixed models when necessary to address confounding factors. The change in LEDD from baseline at each follow-up was included as a covariate in every model. For significant interactions, estimated marginal means are provided in Supplementary Table 3. Significance was defined as *p*-values less than or equal to 0.05. All reported *p*-values are adjusted with false discovery rate correction unless otherwise specified. PDQ scores more than three standard deviations away from the mean were removed from each bin. All analyses were completed using R 3.5.2. Unless otherwise specified, data are presented as mean ± one standard error.

### Reporting summary

Further information on experimental design is available in the Nature Research Reporting Summary linked to this article.

## Supplementary information

Supplemental Materal

Reporting Summary

## Data Availability

The authors confirm that the data supporting the findings of this study are available upon request and subsequent approval from the INFORM database.

## Code availability

The authors confirm that code is available upon request from the corresponding author.
