# Supplementary material for: Long-term Parkinson’s disease quality of life after staged DBS: STN vs GPi and first vs second lead
Source: NPJ Parkinsons Dis. 2020 Jul 6;6:13. doi: 10.1038/s41531-020-0115-3 (PMC7338364; doi:10.1038/s41531-020-0115-3)
Supplement: Supplementary file 1 — Supplemental Materal [file 41531_2020_115_MOESM1_ESM.pdf]

Supplementary Table 1 Baseline characteristics for unilateral patients[a]

Variable (mean±SD)	STN (n=70)	GPi (n=121)	STN vs. GPi
Age	65.07±7.88 (n=70)	66.09±7.71 (n=120)	*p*=0.39, *t*=-0.87
Age at Disease Onset	53.57±10.23 (n=65)	54.08±9.40 (n=102)	*p*=0.99, *W*=3313
Disease Duration	11.51±6.20 (n=65)	11.83±5.69 (n=102)	*p*=0.72, *W*=3205
Gender (male no/no.)	57/70	85/120	*p*=0.15, X^2=2.09
UPDRS-I	2.81±2.35 (n=63)	2.49±1.98 (n=108)	*p*=0.53, *W=3597*
UPDRS-II	15.91±5.96 (n=64)	15.73±6.17 (n=111)	*p*=0.85, *t=-0.19*
UPDRS-III Off-med	37.34±11.78 (n=65)	38.27 ±11.03 (n=114)	*p*=0.60, *t*=-0.52
UPDRS-III Off-med Contralateral Tremor	4.15±2.46 (n=65)	3.09±2.16 (n=114)	***p*<0.01, *W*=2725**
UPDRS-III Off-med Contralateral Rigidity	3.45±1.53(n=65)	3.57±1.44 (n=114)	*p*=0.77, *W*=3608
UPDRS-III Off-med Contralateral Bradykinesia	7.52±2.62 (n=65)	7.5±2.56 (n=114)	*p*=0.96, *W*=3689
UPDRS-III Off-med PIGD	4.29±2.62 (n=65)	5.42±3.07 (n=114)	***p*<0.01, *W*=2828**
LEDD	1439±936 (n=68)	1407±822 (n=118)	*p*=0.85, *W*=3947
Berg Balance Scale	51.65±2.91 (n=26)	50.30±5.29 (n=73)	*p*=0.37, *W*=836
TUG	9.55±1.83 (n=31)	10.89±11.83 (n=79)	*p*=0.55, *W*=1315
Dopamine Responsiveness	-32.32±23.89 (n=62)	-36.24±18.03 (n=109)	*p*=0.46, *W*=3607
MMSE	28.04±2.10 (n=24)	28.67±1.73 (n=30)	*p*=0.19, *W*=432
SWAL-QOL	85.10±10.91 (n=19)	80.56±11.27 (n=24)	*p*=0.17, *W=171*
Anxiety	17.67±7.04 (n=12)	15±9.53 (n=40)	*p*=0.24, *W*=185
Depression	10.52±6.43 (n=31)	11.04±7.45 (n=76)	*p*=0.95, *W*=1188
Total PDQ	30.80±15.25	31.73±15.22	*p*=0.47, *W*=4500
Mobility	36.34±23.02	44.49±27.89	***p*<0.05, *W*=4978**
ADL	39.29±21.41	36.00±21.66	*p*=0.32, *W*=3871
Emotional well-being	29.46±19.21	30.11±20.22	*p*=0.85, *W*=4303
Stigma	30±26.03	25.57±22.20	*p*=0.34, *W*=4584
Social support	10.65±13.69	15.98±19.58	*p*=0.17, *W*=4721
Cognition	28.31±18.33	29.11±20.14	*p*=0.95, *W*=4214
Communication	27.02±21.31	29.99±21.16	*p*=0.34, *W*=4583
Bodily discomfort	45.36±22.04	42.29±22.84	*p*=0.44, *W*=3952

[a]Bold values represent significant differences

Supplementary Table 2 Baseline characteristics for bilateral patients[a]

Variable (mean±SD)	Bilateral First Lead			Bilateral Second Lead			Bilateral First vs. Second	
	STN (n=67)	GPi (n=60)	STN vs. GPi	STN (n=84)	GPi (n=72)	STN vs. GPi	STN	GPi
Age	61.91±8.82 (n=67)	63.95±8.56 (n=60)	*p*=0.19, *t*=-1.32	62.73±9.53 (n=84)	64.79±8.37 (n=72)	*p*=0.24, *W*=2692	*p*=0.42, *W*=2596	*p*=0.57, *t*=-0.57
Age at Disease Onset	50.46±9.10 (n=63)	50.31±9.54 (n=49)	*p*=0.93, *t*=0.09	50.87±9.63 (n=77)	50.42±9.45 (n=59)	*p*=0.78, *t*=0.27	*p*=0.80, *t*=-0.26	*p*=0.95, *t*=-0.06
Disease Duration	11.11±4.59 (n=63)	12.39±4.91 (n=49)	*p*=0.19, *W=1318*	11.83±4.54 (n=77)	13.49±5.15 (n=59)	*p*=0.052, *t=-1.96*	*p=0.27, W=2163*	*p*=0.26, *t*=-1.14
Months between surgeries	-	-	-	14.75±13.30 (n=84)	11.87±11.01 (n=72)	*p*=0.27, *W*=3332	-	-
Gender (male no/no.)	52/67	46/60	*p*=0.90, X^2=0.02	65/84	43/72	*p*=0.58, X^2=0.30	*p*=0.97, X^2=0.001	*p*=0.69, X^2=0.16
LEDD	1572±1442.78 (n=67)	1343.4±732.82 (n=60)	*p*=0.46, *W*=1858	1119±755.55 (n=84)	1559±1921.08 (n=72)	***p*<0.05, *W*=2315**	***p*<0.01, *W*=2083**	*p*=0.66, *W*=2257
UPDRS-I	2.95±2.05 (n=60)	2.25±1.93 (n=56)	*p*=0.054, *W*=2025	2.43±1.80 (n=80)	2.11±1.81 (n=69)	*p*=0.15, *W*=3133	*p*=0.15, *W*=2735	*p*=0.71, *W*=2007
UPDRS-II	15.95±5.91 (n=59)	16.66±5.54 (n=56)	*p*=0.51, *t*=-0.67	13.28±5.52 (n=79)	14.36±5.33 (n=69)	*p*=0.23, *t*=-1.21	***p*<0.01, *t*=2.70**	***p*<0.05, *t*=2.35**
UPDRS-III Off-med	41.17±11.91 (n=58)	40.71±10.94 (n=59)	*p*=0.83, *t*=0.22	38.54±10.80 (n=59)	40.67±10.63 (n=58)	*p*=0.19, *W*=1468	*p*=0.15, *W*=1977	*p*=0.98, *t*=0.02
UPDRS-III Off-med Contralateral Tremor	4.00±2.58 (n=58)	3.49±2.41 (n=59)	*p*=0.31, *W*=1896	3.44±2.48 (n=59)	2.66±2.50 (n=58)	*p*=0.070, *W*=2041	*p*=0.24, *W*=1925	***p*<0.05, *W*=2080**
UPDRS-III Off-med Contralateral Rigidity	3.71±1.53 (n=58)	3.36±1.48 (n=59)	*p*=0.30, *W*=1896	3.07±1.40 (n=59)	3.84±1.71 (n=58)	***p*<0.05, *W*=1282**	***p*<0.05, *W*=2139**	*p*=0.24, *W*=1499
UPDRS-III Off-med Contralateral Bradykinesia	8.05±2.69 (n=58)	7.59±2.61 (n=59)	*p*=0.32, *W*=1531	7.47±2.43 (n=59)	8.00±2.82 (n=58)	*p*=0.28, *t*=1.08	*p*=0.23, *t*=1.22	*p*=0.46, *W=1576*
UPDRS-III Off-med PIGD	4.07±2.38 (n=58)	5.44±3.02 (n=59)	***p*<0.05, *W*=1262**	4.00±2.26 (n=59)	5.79±3.08 (n=58)	***p*<0.001, *W*=2364**	*p*=0.84, *W*=1749	*p*=0.53, *W*=1597
Berg Balance Scale	52.58±4.1 (n=12)	48.67±6.39 (n=30)	***p*<0.05 *W*=258**	50.08±7.27 (n=26)	49.51±5.15 (n=39)	*p*=0.19, *W*=409	*p*=0.23, *W*=195	*p*=0.79, *W*=563
TUG	8.54±2.60 (n=14)	9.22±4.08 (n=31)	*p*=0.71, *W*=202	12.62±15.17 (n=25)	9.96±3.87 (n=45)	*p*=0.57, *W*=609	*p*=0.43, *W*=147	*p*=0.15, *W*=562
Dopamine Responsiveness	-39.11±22.31 (n=57)	-34.45±19.51 (n=57)	*p*=0.08, *W*=1310	-30.99±18.04 (n=54)	-30.05±17.48 (n=53)	*p*=0.78, *t*=-0.27	***p*<0.01, *W*=1099**	*p*=0.21, *t*=-1.25
MMSE	29.05±0.97 (n=21)	27.75±3.82 (n=20)	*p*=0.50, *W*=236	-	-	-	-	-

SWAL-QOL	85.80±9.58 (n=10)	86.74±7.62 (n=17)	p=0.79, t=-0.26	88.86±10.86 (n=14)	83.95±10.21 (n=21)	p=0.08, W=95	p=0.34 W=53	p=0.60, W=197
Anxiety	12±8.09 (n=23)	13.25±9.05 (n=12)	p=0.98, t=-0.02	12.29±8.05 (n=66)	15.00±9.41 (n=54)	p=0.12, W=1486	p=0.58, W=700	p=0.59, W=357
Depression	10.79±6.46(n=14)	8±6.91 (n=36)	p=0.44 W=289	7.79±6.19 (n=34)	8.41±6.57 (n=56)	p=0.64, W=1009	p=0.10, W=166	p=0.45, W=913
Total PDQ	31.72±14.87	32.79±14.68	p=0.69, t=-0.41	23.70±12.51	25.87±11.97	p=0.18, W=2647	**p<0.001, W=1919**	**p<0.01, W=1540**
Mobility	40.82±26.76	49.12±25.58	p=0.06, W=2397	34±24.14	39.70±21.12	p=0.08, W=2538	p=0.13, W=2405	**p<0.05, W=1680**
ADL	43.66±23.66	41.60±23.01	p=0.64, W=2106	29.94±18.59	31.80±18.65	p=0.47, W=3226	**p<0.001, W=3805**	**p<0.05, W=2700**
Emotional well-being	30.85±22.41	28.02±21.08	p=0.52, W=2143	20.41±16.80	19.65±16.67	p=0.88, W=2983.	**p<0.01, W=2052**	**p<0.05, W=1691**
Stigma	26.26±23.35	24.48±22.64	p=0.65, W=1916	12.95±14.93	19.75±18.56	**p<0.05, W=2369**	**p<0.001, W=3823**	p=0.33, W=2373
Social support	16.48±19.33	14.51±19.55	p=0.39, W=2181	9.97±14.58	11.46±17.28	p=0.51, W=3196	**p<0.05, W=3459**	p=0.34, W=2358
Cognition	28.17±17.82	27.55±18.44	p=0.69, W=2094	22.92±15.48	21.40±14.86	p=0.53, W=3200	**p<0.05, W=3345**	p=0.07, W=2559
Communication	28.54±18.67	29.86±22.01	p=0.97, W=2001	24.31±17.85	22.86±17.09	p=0.65, W=3150	p=0.16, W=3190	p=0.11, W=2508
Bodily discomfort	38.99±22.64	47.01±21.55	**p<0.05, W=1574**	31.80±22.67	39.41±20.73	**p<0.01, W=2291**	**p<0.05, W=3428**	**p<0.05, W=2641**

[a]Bold values represent significant differences

Supplementary Table 3 Confidence intervals of estimated marginal means for significant interactions

Model	PDQ Score	Model Variable	Interaction (x vs. y)	x	y
Unilateral	ADL	PIGD	STN vs. GPi	0.46 – 3.99	-2.54– -0.21
Bilateral	Total	LEDD	First vs. Second	0.03 – 0.15	0.005 – 0.04
Bilateral	Total	LEDD	STN vs. GPi	0.04 – 0.12	0.002 – 0.06
Bilateral	Total	Tremor	First vs. Second	-4.48 – 13.57	-12.60 – 0.91
Bilateral	Mobility	Rigidity	STN vs. GPi	-13.41 – 0.49	-1.82 – 5.23
Bilateral	Mobility	LEDD	First vs. Second	0.001 – 0.049	-0.008 – 0.008
Bilateral	ADL	LEDD	First vs. Second	0.0003 – 0.038	-0.008 – 0.005
Bilateral	ADL	Baseline score	First vs. Second	-1.23 – -0.53	-0.50 – 0.005
Bilateral	Emotional well-being	Baseline score	First vs. Second	-0.88 – -0.38	-0.47 – 0.005
Bilateral	Emotional well-being	Tremor	First vs. Second	0.002 – 4.94	-3.6 – 0.13
Bilateral	Cognition	Baseline score	First vs. Second	-0.92 – -0.42	-0.15 – 0.30

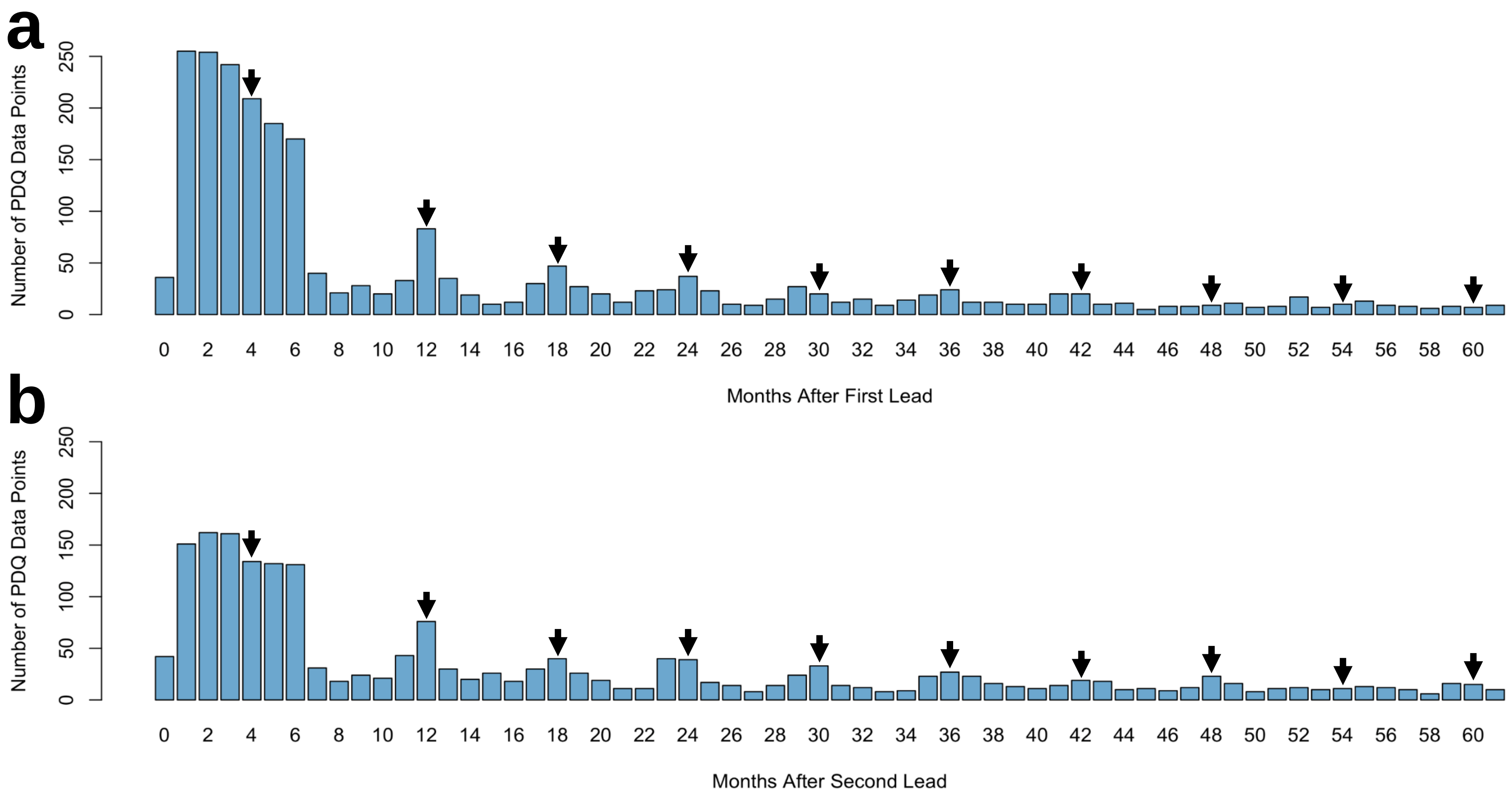

Supplementary Figure 1. The number of PDQ time points in our retrospective database. The first lead (a) includes unilateral leads and the first lead for bilateral patients. The second lead (b) only represents the second lead of bilateral patients. There are peaks seen at 4, 12, 18, 24, 30, 36, 42, 48, 54, and 60 months, which are denoted by arrows.
